# Knowledge, attitude, and practice regarding cardiovascular diseases in Saudi Arabia: A cross-sectional study

**DOI:** 10.1097/MD.0000000000041597

**Published:** 2025-02-28

**Authors:** Shalan Alaamri, Abdallah Y. Naser

**Affiliations:** a Department of Medicine, College of Medicine, University of Jeddah, Jeddah, Saudi Arabia; b Department of Applied Pharmaceutical Sciences and Clinical Pharmacy, Faculty of Pharmacy, Isra University, Amman, Jordan.

**Keywords:** attitude, cardiovascular diseases, knowledge, practice, Saudi Arabia

## Abstract

Cardiovascular diseases (CVDs) are the primary contributors to mortality and morbidity on a global scale. The aim of this study is to examine knowledge, attitude and practices of the general public regarding CVDs in Saudi Arabia. This is a cross-sectional survey study that was conducted in Saudi Arabia to examine KAP of the general public regarding CVDs between January and February 2024. There were 605 participants in total enrolled in this study. The mean knowledge score for the study participants was 7.1 (2.5) out of 11 (64.5%); which indicates moderate level of knowledge of CVD. A considerable proportion of participants (61.5%) report engaging in vigorous physical activity for a minimum of 30 minutes per day throughout the week, including activities like running, lifting large loads, or drilling. In terms of knowledge, there is no significant difference between males and females across gender categories (odds ratio = 0.92, *P* = .603). Within the age group category, those between the ages of 24 and 30 (odds ratio = 0.44, *P* < .001) and 31 and 40 (odds ratio = 0.39, *P* < .001) demonstrate noticeably diminished probabilities of possessing superior knowledge in comparison to those between the ages of 18 and 23. In relation to marital status, the odds of possessing superior knowledge are considerably lower for married individuals (odds ratio = 0.43, *P* < .001), divorced individuals (odds ratio = 0.39, *P* = .003), and widowed individuals (odds ratio = 0.11, *P* = .004) in comparison to singles. The survey found moderate level of awareness of cardiovascular diseases and strong recognition of preventive measures including physical activity and a healthy diet. There are gaps in CVD symptom knowledge. Participants are typically favorable about CVD prevention and willing to adopt healthy lifestyles. Decision-making recommendations include targeted teaching efforts on symptom awareness, physical activity, and healthy diets. Further research is needed to determine how educational interventions improve knowledge across demographic groups. Longitudinal studies could also evaluate lifestyle treatments for CVD risk reduction.

## 1. Introduction

Cardiovascular diseases (CVDs) are the primary contributors to mortality and morbidity on a global scale,^[1]^ resulting in approximately 17.9 million deaths annually. It is worth noting that over 75% of these deaths occur in countries with lower economic status.^[2]^ In 2016, the number of Saudi citizens affected by CVD was 201,300. This included 149,600 adults diagnosed with ischemic heart disease and 51,700 individuals with cerebrovascular illness. CVD is responsible for more than 45% of all deaths, according to estimates.^[3,4]^ The estimated prevalence of coronary artery disease (CAD) in Saudi Arabia in 2017 was 1.6%.^[5]^ Given the increase in risk factors such as obesity, dyslipidemia, hypertension, diabetes mellitus, and others, it is anticipated that the present prevalence of CVD in Saudi Arabia will be considerably higher.^[6]^ The healthcare expenditure in Saudi Arabia accounts for a collective burden exceeding 8% (or an estimated $56 billion) of the government’s overall budget.^[7]^ The economic impact of CVD in Saudi Arabia was assessed at $3.5 billion in 2016, comprising $1.9 billion in direct healthcare expenses and $1.6 billion in indirect costs resulting from decreased productivity. By 2035, this amount is projected to triple to $9.8 billion.^[4]^ It has long been recognized that promoting healthy behaviors is necessary to prevent the spread of vascular diseases, including CVDs.

Given that the majority of risk factors for CVD may be prevented or managed, including smoking, obesity, unhealthy diets, lack of physical activity, hypertension, diabetes, and dyslipidemia, it is crucial to implement targeted population-based prevention programs to effectively prevent these diseases.^[8]^ While CVDs are influenced by multiple factors, both individual and socio-environmental, research suggests that a higher level of knowledge in health-related matters might lead to changes in attitude. Over time, it leads to noticeable changes in behavior.^[9]^ Hence, it is clear that a crucial requirement for altering health behaviors and lifestyles is enhancing the understanding of CVD and its alterable risk factors.^[10,11]^ Knowledge, attitude, and practice (KAP) surveys are beneficial for evaluating the success of intervention programs. This is important for public health in order to build specific educational programs. The aim of this study is to examine KAP of the general public regarding CVDs in Saudi Arabia.

## 2. Method

### 2.1. Study design

This is a cross-sectional survey study that was conducted in Saudi Arabia to examine KAP of the general public regarding CVDs between January and February 2024.

### 2.2. Sampling procedure

The study sample was collected using a technique known as convenience sampling. This sampling method is a type of non-probability sampling. This study involved eligible people who satisfy our inclusion criteria and are willing to participate due to their availability. The first page of the questionnaire displayed a consent form, providing participants with the option to continue or terminate their participation. In order to ensure that participants fully comprehend the importance of their involvement, the study’s objectives were clearly stated. The study’s invitation letter included a clear overview of the inclusion criteria.

### 2.3. Population studied

Invitations were extended to all individuals presently residing in Saudi Arabia to take part in this study. The URL for the questionnaire were disseminated through several social media platforms, including Facebook, Snapchat, and Instagram. Eligibility was limited to individuals who currently reside in Saudi Arabia and have reached the age of 18 or older. There are no limitations based on gender, occupation, or nationality.

### 2.4. Study tool

This study utilized a previously developed questionnaire by Koohi et al to assess the KAP of participants regarding CVD.^[12]^ The original questionnaire tool was developed through a process consisting of 5 stages: item production, face validity, content validity, construct validity, and reliability. The relevant elements were derived from the outcomes of a comprehensive literature research and a semi-structured interview with 5 experts in the fields of health education, epidemiology, cardiovascular disease, and nutrition. To begin with, a comprehensive review of existing literature on CVD knowledge, attitude, and practice was performed. This search aimed to identify suitable items for the study instrument using specific keywords such as “cardiovascular disease,” “CVD,” “questionnaire,” “scale,” “knowledge,” “attitude,” “practice,” “behavior,” and “KAP” in popular English databases including PubMed, Scopus, and Google Scholar.

The questionnaire tool comprised 5 sections: the first section examined participants’ demographic characteristics; section 2 contained 12 valid and reliable items measuring knowledge, with total raw scores ranging from 0 to 24; section 3 consisted of ten items measuring attitude, with total raw scores for attitude ranging from 10 to 50; section 4 included 2 items measuring physical activity behaviors; and finally, section 5 encompassed 4 items measuring nutrition and smoking behaviors.

### 2.5. Questionnaire translation

The questionnaire tool was translated to Arabic language using forward-backward translation technique. This was conducted in order to facilitate the participation of the general public in the study.

### 2.6. Questionnaire piloting

The questionnaire instrument was reviewed and verified by 2 qualified physicians who assessed its clarity and readability, including its face validity and the comprehensibility of each item. Structured feedback was provided on the questionnaire tool, which was deemed appropriate and clear. Therefore, no changes were required to be made. Furthermore, before implementing the questionnaire on a larger scale, a pilot study on 40 participants from the target study population was carried out to evaluate its comprehensibility. Cronbach alpha reliability coefficient for the questionnaire tool was 0.72 which deemed acceptable.

### 2.7. Sample size

Using a 95% confidence interval, a standard deviation (SD) of 0.5, and an error margin of 5%, the minimum required sample size is 385 individuals.

### 2.8. Statistical analysis

Version 29 of Statistical Package for Social Science (Chicago) was used to conduct the statistical analysis. Using descriptive statistics, the demographic characteristics of the participating individuals were described. The normality of the continuous variable was examined using histogram and normality measures. On this basis, continuous variables, such as the knowledge score of the participants was depicted using the mean and SD for normally distributed data. We used percentages (frequencies) to report categorical data. Binary logistic regression analysis was used to identify predictors of being knowledgeable of CVDs. Multivariate analysis was conducted to adjust for potential confounding variables, which are gender, age, marital status, education level, monthly income level, employment status, and comorbidities history. The cutoff point used to identify the dummy variable for knowledge regression model was assigned based on the study participant’s mean knowledge score (which is 7.1 [SD: 2.5]). Statistical significance was defined as a 2-sided *P*-value less than or equal to .05.

## 3. Results

There were 605 participants in total enrolled in this study. A comprehensive breakdown of the demographic and socioeconomic characteristics of the surveyed population is presented in Table 1. With regard to the distribution of genders, females comprise the plurality at 54.4%. The data reveals that a significant percentage of participants fell within the age range of 24 to 30 years, constituting 43.1% of the sample. The age group of 31 to 40 years follows with 25.3% of the respondents. In relation to the respondents’ marital status, a considerable proportion are married (59.2%), whereas the unmarried population comprises 29.3%. The bachelor’s degree level comprises the majority of educational attainment (60.2%), while secondary school or lesser education comprises a smaller proportion (6.1%). The monthly income distribution indicates that a significant proportion of individuals (45.3%) earn an income exceeding 7500 Saudi Arabian Riyals. The employment status of individuals reveals a wide range of perspectives, as the majority (57.5%) are employed in sectors other than healthcare, while the unemployed comprise the remaining 14.0%. Additionally, a significant percentage of participants (36.4%) disclose having concurrent medical conditions.

**Table 1:** Participants’ demographic characteristics.

Variable	Frequency	Percentage
Gender		
Females	329	54.4
Age groups		
18 to 23 yr	131	21.7
24 to 30 yr	261	43.1
31 to 40 yr	153	25.3
41 to 50 yr	35	5.8
51 to 60 yr	18	3.0
61 yr and older	7	1.2
Marital status		
Single	177	29.3
Married	358	59.2
Divorced	54	8.9
Widowed	16	2.6
Education level		
Secondary school level or lower	37	6.1
Diploma	143	23.6
Bachelor’s degree level	364	60.2
Higher education level	61	10.1
Monthly income level		
<2500 SAR	59	9.8
2500 to 5000 SAR	118	19.5
5001 to 7500 SAR	154	25.5
>7500 SAR	274	45.3
Employment status		
Retired	14	2.3
Unemployed	85	14.0
Work in the healthcare sector	75	12.4
University student (medical field)	30	5.0
Work outside the healthcare sector	348	57.5
University student (nonmedical field)	53	8.8
Have comorbidities: (yes)	220	36.4

### 3.1. Knowledge of cardiovascular diseases

The mean knowledge score for the study participants was 7.1 (SD: 2.5) out of 11 (64.5%); which indicates moderate level of knowledge of CVD. The responses of the participants concerning their understanding of cardiovascular diseases are presented in Table 2. The preponderance of participants recognizes the prophylactic advantages of engaging in physical activity (61.5%) and the favorable influence of incorporating fruits and vegetables into their daily diets on cardiovascular well-being (64.6%). Additionally, a notable percentage of respondents acknowledge the presence of a family history of cardiovascular disease as a risk factor (60.8%), in addition to the correlation that exists between overweight or obesity and heightened cardiovascular risk (73.4%). Significantly high levels of awareness (74.9%) exist regarding the detrimental impacts of tobacco use on cardiovascular health. Furthermore, a significant majority recognizes the adverse effects of consuming canned and salted foods on blood pressure (77.4%). Furthermore, they emphasize the criticality of managing hypertension and hyperglycemia to prevent cardiovascular complications (62.5% and 61.5%, respectively). On the other hand, there seems to be a relatively diminished level of knowledge concerning particular indications of cardiovascular incidents, such as sudden numbness or weakness in the muscles of the face, limbs, or legs (49.1%), which could be a sign of a stroke or chest attack, respectively (58.8% and 49.1%, respectively).

**Table 2:** Participants’ response to items related to knowledge of cardiovascular diseases.

Variable	Frequency (%)
Physical activity can prevent cardiovascular disease.	372 (61.5)
Daily eating of fruits and vegetables has a beneficial effect on cardiovascular health.	391 (64.6)
The history of cardiovascular disease in the family (father, mother, sister, or brother) can increase the risk of cardiovascular disease	368 (60.8)
There is a higher risk of cardiovascular disease in people who are overweight or obese.	444 (73.4)
Using tobacco (cigarettes, hookah, pipe, etc) can increase the risk of cardiovascular disease.	453 (74.9)
Consumption of salty and canned foods increases the risk of rising blood pressure.	468 (77.4)
Controlling blood glucose and the prevention of diabetes can reduce the risk of cardiovascular complications	378 (62.5)
Controlling high blood pressure is vital to prevent myocardial infarction	372 (61.5)
Feeling of pain, pressure, or burning in the chest can be a symptom of a heart attack	356 (58.8)
Feeling of pain or sudden discomfort in the jaw, neck, between the 2 scapulas, shoulders, or arms and stomach area can be a symptom of a heart attack.	297 (49.1)
Sudden numbness or weakness in the face, arms, or legs muscles can be signs of a stroke.	371 (49.1)

### 3.2. Attitude towards cardiovascular diseases

The attitudes of the participants with regard to cardiovascular diseases are detailed in Table 3. A substantial majority of participants (70.2%) confirmed their conviction regarding the criticality of physical activity in sustaining a healthful way. Additionally, for short distances, walking is preferred to motorized transportation (64.3%). The prevalence of awareness concerning the detrimental consequences of tobacco use was confirmed by 67.3% of individuals. Furthermore, a notable proportion exists to embrace health-conscious dietary practices as 66.6% consume fewer oily foods, 64.0% believe in the advantages of maintaining a healthy weight, and 62.6% incorporate fruits and vegetables into their diets on daily basis. The participants also demonstrate an understanding of the significance of managing particular risk factors, including blood glucose control to prevent myocardial infarction (64.0%) and stress management for heart health (62.0%). Furthermore, 70.9% of respondents acknowledge the critical nature of sodium restriction. However, 59.7% of them recognized the potential advantages of consistent fish intake in terms of cardiovascular well-being.

**Table 3:** Participants’ response to items related to attitude towards cardiovascular diseases.

Variable	Frequency (%)
I believe that I should have physical activity to have a healthy life.	425 (70.2)
I believe that I should try to walk to go to nearer destinations instead of going by taxi or bus.	389 (64.3)
I believe that using any tobacco (cigarette, hookah, pipe, etc) is harmful to health.	407 (67.3)
I believe that having an appropriate weight (not overweight or obesity) helps keep me healthy.	387 (64.0)
I believe that I must consume less fatty foods to maintain health	403 (66.6)
I believe that daily consumption of 2 to 4 units of fruit and 3 to 5 units of raw or cooked vegetables is beneficial for my health.	379 (62.6)
I believe that uncontrolled blood glucose in diabetic patients can cause myocardial infarction.	387 (64.0)
I believe that I should control my stress and mental pressure to prevent myocardial infarction.	375 (62.0)
I believe that I should consume less salt to prevent high blood pressure.	429 (70.9)
I believe that consuming fish meat at least 2 times a wk is beneficial for cardiovascular health	361 (59.7)

### 3.3. Practices related to cardiovascular diseases

The practices of the participants with regard to cardiovascular health are shown in Table 4. A considerable proportion of participants (61.5%) report engaging in vigorous physical activity for a minimum of 30 minutes per day throughout the week. Furthermore, a considerable percentage of participants engage in moderate physical activity for the same duration, such as transporting light loads or walking quickly, as reported by 59.2% of the respondents.

**Table 4:** Participants’ response to items related to practices related cardiovascular diseases.

Variable	Frequency (%)
Do you have intense physical activity such as running, carrying heavy loads, drilling, and... at least 30 min a day during the wk?	372 (61.5)
Do you have moderate physical activity such as fast walking or carrying light loads at least 30 min a day during the wk?	358 (59.2)

### 3.4. Predictors of better knowledge of CVD

Table 5 presents predictors of better knowledge of CVD. Within the age group category, those between the ages of 24 and 30 (odds ratio = 0.44 [95% CI = 0.29–0.68), *P* < .001) and 31 and 40 (odds ratio = 0.39 [95% CI = 0.24–0.62], *P* < .001) demonstrate noticeably diminished probabilities of possessing superior knowledge in comparison to those between the ages of 18 and 23. In relation to marital status, the odds of possessing superior knowledge are considerably lower for married individuals (odds ratio = 0.43 [95% CI = 0.30–0.62], *P* < .001), divorced individuals (odds ratio = 0.39 [95% CI = 0.20–0.73], *P* = .003), and widowed individuals (odds ratio = 0.11 [95% CI = 0.02–1.23], *P* = .004) in comparison to singles. Knowledge level was not significantly predicted by gender, monthly income, education level, employment status. Having comorbidities history raised the likelihood of better knowledge level with odds ratio is 1.48 (95% CI = 1.05–2.09); (*P* = .024). Multivariate analysis identified that married and widowed participants were less likely to be knowledgeable of CVD compared to others (*P* < .05).

**Table 5:** Predictors of better knowledge of cardiovascular diseases.

Variable	Unadjusted univariate logistic regression analysis		Multivariate regression analysis	
	Odds ratio of having better knowledge (95% confidence interval)	*P*-value	Odds ratio of having better knowledge (95% confidence interval)	*P*-value
Gender				
Females (reference category)	1.00		1.00	
Males	0.92 (0.66–1.27)	.603	1.10 (0.75–1.60)	.625
Age groups				
18 to 23 yr (reference category)	1.00		1.00	
24 to 30 yr	0.44 (0.29–0.68)	<.001	0.69 (0.34–1.35)	.300
31 to 40 yr	0.39 (0.24–0.62)	<.001	0.67 (0.31–1.43)	.847
41 to 50 yr	0.82 (0.39–1.72)	.593	1.11 (0.39–3.07)	.980
51 yr and older	0.51 (0.22–1.23)	.134	1.02 (0.29–3.53)	.641
Marital status				
Single (reference category)	1.00		1.00	
Married	0.43 (0.30–0.62)	<.001	0.56 (0.33–0.94)	.031*
Divorced	0.39 (0.20–0.73)	.003	0.56 (0.26–1.19)	.128
Widowed	0.11 (0.02–0.50)	.004	0.14 (0.03–0.69)	.019*
Education level				
Secondary school level or lower (reference category)	1.00		1.00	
Diploma	1.24 (0.61–2.55)	.554	1.84 (0.78–4.34)	.165
Bachelor’s degree level	1.77 (0.75–4.15)	.190	2.04 (0.74–5.64)	.169
Higher education level	2.11 (0.99–4.53)	.054	1.67 (0.72–3.83)	.231
Monthly income level				
<2500 SAR (reference category)	1.00		1.00	
2500 to 5000 SAR	0.73 (0.39–1.38)	.334	1.35 (0.65–2.92)	.436
5001 to 7500 SAR	0.79 (0.43–1.44)	.437	1.70 (0.80–3.69)	.174
>7500 SAR	0.73 (0.42–1.29)	.278	1.85 (0.86–3.85)	.108
Employment status				
Retired (reference category)	1.00		1.00	
Unemployed	0.89 (0.28–2.79)	.840	0.89 (0.21–3.74)	.875
Work in the healthcare sector	1.70 (0.54–5.37)	.368	1.69 (0.41–7.03)	.472
University student (medical field)	1.74 (0.48–6.28)	.395	1.56 (0.32–7.57)	.581
Work outside the healthcare sector	0.64 (0.22–1.89)	.421	0.68 (0.18–2.61)	.570
University student (nonmedical field)	2.82 (0.85–9.43)	.091	2.08 (0.44–9.80)	.353
Have comorbidities				
No (reference category)	1.00			
Yes	1.48 (1.05–2.09)	.024*	0.91 (0.61–1.37)	.656

* *P* < .05.

## 4. Discussion

Based on the escalating rate of CVD risk factors in Saudi Arabia, such as hypertension, dyslipidaemia, diabetes mellitus, obesity, and others, it is predicted that the existing prevalence of CVDs in the country has substantially risen.^[1]^ Several factors have increased the risk of CVDs in different areas, including Saudi Arabia, including socioeconomic status, lifestyle changes, and urbanization.^[13]^ Measuring public KAP concerning CVD is important to develop and implement adequate CVD management and preventive intervention programs. Our aim in this study was to assess the level of KAP on CVD and identify factors associated with better CVD understanding among the public in Saudi Arabia.

Numerous prior studies have demonstrated a lack of and low levels of knowledge regarding CVD among the public in Saudi Arabia, including studies in Saudi Arabia,^[14]^ in the South region of Saudi Arabia,^[15]^ in the western region of Saudi Arabia,^[16]^ and Jeddah City.^[17]^ However, our study revealed a moderate level of knowledge (64.5%) regarding CVD among public participants in Saudi Arabia. This indicates a potential improvement in public health knowledge concerning CVD in Saudi Arabia.

Moreover, our findings of moderate knowledge level appeared relatively better when compared with findings from studies in other countries. Prior studies in Iraq,^[18]^ Jordan,^[19]^ Kuwaiti,^[20]^ and Lebanon^[21]^ have demonstrated deficiencies in knowledge levels regarding CVD. Additionally, an earlier meta-analysis across various countries has indicated restricted knowledge about CVD and its risk factors among adults aged 18 to 34 years.^[22]^ A previous study revealed considerable differences in knowledge about CVD, especially CVD risk factors and symptoms, among individuals.^[23]^ Differences in knowledge between countries could also be influenced by social, economic, cultural, and epidemiological factors. However, public efforts and interventions to address these aspects can help increase knowledge and awareness within these regions and globally.

Our findings also align with previous research indicating moderate CVD knowledge levels among the Najran population in Saudi Arabia.^[24]^ However, the persistent moderate knowledge, in addition to the increased prevalence of CVD in Saudi Arabia, suggests that there remains a need to enhance public knowledge and understanding of CVD. Thus, implementing targeted interventions to enhance public knowledge of CVD in Saudi could improve CVD health outcomes within populations.

The bulk of health behavior decisions are influenced by people’s understanding and thoughts of risk factors for their diseases.^[25]^ Therefore, although disease awareness alone is inadequate for sufficient healthcare consequences, research consistently highlights the crucial role of disease awareness in assisting people in implementing better healthcare decisions.^[25,26]^ In our study, we found that there is widespread recognition of the importance of physical activity, healthy dietary practices, and awareness of risk factors like family history, obesity, and tobacco use. However, our findings also indicated a relatively lower awareness regarding specific symptoms of cardiovascular events (sudden numbness or weakness in the muscles of the face, limbs, or legs, which could be a sign of a stroke or chest attack). However, a prior study conducted among the general population in Saudi Arabia in 2016 highlighted insufficient knowledge regarding CVD risk factors,^[27]^ indicating potential improvement in awareness regarding CVD risk factors among the public and disparities in awareness across different demographics. Aligns with our findings, previous studies conducted in Kuwait^[20]^ and Buea, Cameroon,^[28]^ demonstrated challenges in recognizing specific symptoms of cardiovascular events among the public. These suggest a gap in cardiovascular events knowledge among the public. Targeted educational interventions focusing on distinct symptoms of cardiovascular events could be beneficial in addressing existing knowledge gaps.

The attitudes towards CVD among our participants were generally positive, emphasizing physical activity, healthy dietary choices, and stress management. These positive perceptions suggest that promoting preventive measures and lifestyle modifications will reduce CVD risk, particularly physical inactivity, unhealthy dietary choices, and stress. Previous studies have comparable findings. For instance, a prior meta-analysis revealed inadequate attitudes about CVD and related risk factors among young adults across various countries,^[22]^ highlighting the probability of differences in attitudes across age groups or demographics. Furthermore, studies conducted in Saudi Arabia,^[24]^ Iran,^[29]^ Nepal,^[30]^ Croatia,^[31]^ Lebanon,^[21]^ and rural communities in Lahore^[32]^ reported varying cardiovascular health levels, ranging from perfect attitude levels among the Saudi and Iranian population to restricted attitudes in Lebanon and Croatia and relatively unfavorable attitudes towards CVD risk factors in communities in Lahore. These findings underscore the intricate associations of cultural, regional, and socioeconomic factors in influencing attitudes regarding cardiovascular health. Addressing negative attitudes and encouraging healthier lifestyle choices through tailored interventions are crucial to improving global cardiovascular health.

In our study, we found that practices regarding cardiovascular health demonstrate significant engagement in regular physical activity, both vigorous and moderate. This indicates a positive and promising trend to manage and prevent CVD through adopting healthy lifestyle behaviors. On the contrary, a prior study in Saudi Arabia demonstrated insufficient physical activity levels,^[33]^ indicating a shift towards adopting healthier lifestyle behaviors and managing CVD risk. These could also highlight the effectiveness of targeted interventions in enhancing physical activity among the Saudi population. In line with our findings, recent statistics from the General Authority for Statistics indicate a notable increase in practicing physical activity among adults across Saudi Arabia by about 30%.^[34]^

In our study, we found that predictors of better knowledge include age group, marital status, and the presence of comorbidities. Conversely, gender, education level, monthly income, and employment status showed no significant associations except for individuals with comorbidities, who demonstrated significantly higher knowledge levels. In comparison, previous research in Kuwait established that CVD family history, adherence to a healthy diet, gender (higher among females), level of education, and age groups were significant determines of CVD knowledge.^[20]^ Another prior study revealed that females exhibited higher CVD knowledge among pharmaceutical company workers in Tehran.^[35]^ Furthermore, the Kuwait study,^[20]^ conversely to our findings, indicated a lack of significant knowledge elevation among precipitants with dyslipidaemia, hypertension, diabetes, and CAD. These highlight a gap in knowledge of CVD, particularly among individuals at heightened risk. Again, these findings underscore the importance of targeted interventions tailored to distinct demographics and health status.

Older participants demonstrated lower likelihood of being knowledgeable of CVD. This could be justified that older individuals could have lower level of exposure to educational campaign whether through social media or their work environments. Besides, for elderly individuals declining cognitive function could be another possible contributing factor. Furthermore, in our study, we found that single individuals demonstrated higher level of knowledge compared to others. This can be justified that single individuals are more oriented towards their personal health, self-educating themselves, and engage in preventive measures.

This study has limitations. The use of cross-sectional study design restricted the ability to examine causality among the study variables. Besides, this was an online survey study that was distributed anonymously to the targeted study population. We were not able to estimate the number of participants who received the questionnaire; therefore, we are unable to estimate the response rate. Therefore, our findings should be interpreted carefully.

## 5. Conclusion

This study demonstrates that participants had a moderate degree of awareness regarding CVD, and they strongly acknowledge the importance of preventive measures such as engaging in physical exercise and maintaining a healthy diet. Nevertheless, there remain deficiencies in our understanding of the precise symptoms associated with CVD. Participants often have a positive attitude towards the prevention of CVD and show a readiness to embrace healthy lifestyle habits. Suggestions for decision-making involve implementing focused educational campaigns that emphasize the need of recognizing symptoms and the advantages of engaging in physical exercise and maintaining healthy diets. Additional research is necessary to investigate the efficacy of various educational approaches in enhancing knowledge levels across diverse demographic groups. In addition, longitudinal studies could evaluate the effects of lifestyle changes on decreasing risk factors associated with CVD.

## Author contributions

**Conceptualization:** Shalan Alaamri.

**Data curation:** Shalan Alaamri, Abdallah Y. Naser.

**Formal analysis:** Abdallah Y. Naser.

**Funding acquisition:** Shalan Alaamri.

**Investigation:** Shalan Alaamri, Abdallah Y. Naser.

**Methodology:** Shalan Alaamri, Abdallah Y. Naser.

**Project administration:** Shalan Alaamri.

**Resources:** Shalan Alaamri, Abdallah Y. Naser.

**Software:** Abdallah Y. Naser.

**Supervision:** Shalan Alaamri.

**Validation:** Shalan Alaamri, Abdallah Y. Naser.

**Visualization:** Shalan Alaamri, Abdallah Y. Naser.

**Writing – original draft:** Shalan Alaamri, Abdallah Y. Naser.

**Writing – review & editing:** Shalan Alaamri, Abdallah Y. Naser.
